# Investigating the effect of social networking site use on mental health in an 18–34 year-old general population; a cross-sectional study using the 2016 Scania Public Health Survey

**DOI:** 10.1186/s12889-020-09732-z

**Published:** 2020-11-23

**Authors:** Emily Stella Scott, Catarina Canivet, Per-Olof Östergren

**Affiliations:** Social Medicine and Global Health, Department of Clinical Sciences Malmö, Lund University, Lund, Sweden

**Keywords:** Online social networking, Social media, Mental health, Gender, Young adult, Social support, Perceived emotional support

## Abstract

**Background:**

Social Networking Sites (SNS) are commonly used, especially by young adults. Their impact on mental health is unclear. Moreover, little is known about how social factors, e.g. Perceived Emotional Support (PES), may affect this association. Mental health issues are increasingly burdening the young generation and society as a whole. This study aims to investigate the association between frequency of SNS use and number of SNS contacts with the mental health of a young, Swedish population. Additionally, the potential effect modification of PES will be analysed in regard to these relationships.

**Method:**

This cross-sectional study applied logistic regression analyses to data on 1341 participants (aged 18–34), retrieved from the Scania Public Health Survey (2016). Analyses were stratified by gender and the GHQ-12 scale assessed poor mental health. A 2-way interaction model was used to test for effect modification by PES regarding the association between SNS use and mental health.

**Results:**

Increased risk for poor mental health was found in women only. Using SNS almost hourly vs. less often resulted in an odds ratio (OR) of 1.66 (95% confidence interval (CI) = 1.16–2.38). The corresponding figures for having ≥600 contacts vs. ≤599 were (1.89; 1.21–2.97). Having low PES and using SNS almost hourly was associated with an OR of 3.12 (CI = 1.69–5.76; synergy index (SI) = 1.25). Low PES and ≥ 600 contacts resulted in an OR of 6.07 (CI = 1.73–21.33), whereby interaction was detected (SI = 2.88).

**Conclusion:**

Women, but not men, with frequent SNS use and a high number of SNS contacts were more likely to have poor mental health, which was exacerbated in women with low PES. Facilitating PES could be an approach for improving mental health among young adults. Future studies on the use of SNS should focus more on gender analyses.

**Supplementary Information:**

**Supplementary information** accompanies this paper at 10.1186/s12889-020-09732-z.

## Background

The invention and widespread use of Social Networking Sites (SNS) has arisen alongside the New Media Age. Websites such as Facebook, Instagram or Twitter were designed primarily for communication purposes, where one can instantly message contacts, share photos, videos, or statements [1]; but also for entertainment, socialising or sharing news. As the use of SNSs is still a relatively novel phenomenon, the long- and short-term effect on health, especially mental health, is somewhat unknown and largely controversial [2, 3]. In Sweden, the number of SNS users has risen steadily. In 2017, 81% of the population aged above 12 were using them, 56% on a daily basis; compared to 53 and 28%, respectively, in 2010. Individuals aged between 12 and 35 most commonly use SNS on a daily basis, women more than men. The most commonly used site in Sweden is Facebook, whereby in 2017, 76% of Internet users aged 16–25 and 74% aged 26–35 used Facebook on a daily basis [4].

Mental health problems are on the rise worldwide [5], largely due to an increase in depression [5, 6]. Depressive disorders reside in the top three leading causes of Years Living with Disabilities (YLD), globally [6], with recent estimates predicting around 270 million affected individuals [5]. Depression correlates with an increased mortality, especially suicide [7], which is the second most common cause of death among 15–29-year olds [5]. In Sweden, the level of poor mental health in children and young adults has also risen in recent years, with no apparent aetiology [8]. The parallel rise in SNS use and poor mental health prevalence among young adults may suggest the former is affecting the latter [9].

### Mental health and frequency of SNS use

It has been postulated that the mechanism linking SNS use and mental health issues may be increased time spent on SNS, thereby interfering with routine obligations and functioning [3]. One longitudinal study, using the experience sampling method, showed that increased SNS use predicted declines in affective well-being and life satisfaction [10]. Users may expect to ‘feel better than before’ after using Facebook for about 20 min, according to a three-part study on Facebook’s emotional consequences [11]. However, in an experimental part of this same study, increased time on Facebook prior to an assessment of emotional status correlated with negative mood [11]. Essentially, these results may suggest that if users’ subjective well-being is consistently undermined over a sustained period, this could lead to depression [2].

However, some articles posit that there is no significant effect of frequency of SNS use on depression [12, 13]. Others suggest that certain types of activity and their specific SNS use predict depression. For example, passive SNS use (in the case of Facebook), would include browsing the newsfeed and reading contacts’ posts and profiles. During active Facebook use, one is actively posting content, engaging with other people’s content or communicating [2]. This could be an issue, because in an experimental and experience sampling study, passive Facebook use was shown to decrease affective well-being, whereas active Facebook use did not [14]. Several studies concern themselves with specific negative feelings associated with SNS use, such as envy [14, 15], loneliness [16] or worry [10]. For instance, the relationship between passive Facebook use and affective well-being was mediated by envy, so that passive Facebook use increased feelings of envy which in turn decreased well-being [14]. Similarly, Tandoc et al. (2015) [15] found that depression occurred when envy was triggered during passive Facebook use. If envy did not occur, use of Facebook correlated positively with lower levels of depression [15].

The majority of studies have investigated the use of Facebook [2], though the use of Instagram also seems relevant. In Sweden, Instagram is used by 52% of the population, mostly by younger individuals [4]. Frequency of Instagram use was positively correlated with depressive symptoms, anxiety and self-esteem issues in a cohort study of 129 women between 18 and 35 years of age. These associations were partially mediated by social comparison [17]. Similarly, in another study of a mixed gender population, more frequent use of Instagram was significantly positively associated with depression [18].

### Mental health and the number of SNS contacts

The number of SNS contacts is a frequently studied factor that may influence the relationship between SNS use and mental well-being [2, 3]. Considering the results of the studies from section Mental health and frequency of SNS use, these may suggest that more SNS contacts exposes the individual to more content that could be detrimental to their mental well-being, for example due to a tendency to compare themselves or ruminate. In the Instagram study, the number of accounts participants were following as well as the participants’ number of followers correlated positively with depressive symptoms [17]. A further Instagram inquiry also found a positive association between Instagram use and symptoms of depression when following disproportionately more strangers than real life acquaintances. When following more acquaintances than strangers, increased Instagram use correlated with decreased depression [18]. This study is interesting in that it suggests there is a point at which following too many strangers can be detrimental to one’s well-being. Moreover, having a high number of Facebook friends predicted worsened life satisfaction for those who used Facebook for making new connections as opposed to strengthening current friendships [19]. In contrast, a study with a Finnish population, of whom most were students, concluded that the number of Facebook friends had no association with happiness nor life satisfaction, as this was confounded by personality traits [20]. It seems reasonable to hypothesise that having increasingly more SNS friends increases the likelihood of a larger proportion of these being distant acquaintances, superficial types of relationships or even total strangers, as was revealed in a study of college students’ Facebook friendship networks [21]. This might increase the exposure of other people’s SNS profiles and posts with whom and which one is less familiar with, in turn increasing the chances of using SNS in a less active and more passive way. Alternatively, a higher number of online contacts may, via the same logic, potentially increase negative social comparison, fuelling feelings of envy and thereby contributing to depressive mood [18, 22].

### Perceived emotional support

Perceived emotional support (PES) is defined as subjectively perceived support, which provides empathy or advice during times of trouble from important others, such as family, friends and partners, which differs from received support (the support you *actually* receive) [23]. Having low PES has been associated with increased depression and anxiety [24], poor general health, quality of life and other mental health outcomes [25].

Previously, interest in PES derived online from the use of SNS in association with mental well-being has been far greater, given SNS are social portals and so a potential source of PES [2]. However, it seems no study so far has investigated the role of “offline PES” in the relationship between SNS use and mental health. Understanding people’s environmental social factors such as their level of PES, could yield a better understanding of the associations between SNS use and mental health. In addition, as the above account of studies has shown, it is unlikely to be social media use alone that impacts mental health, but rather a combination with other factors, such as personality and social support structures.

In summary, research into the impact of frequency of SNS use and network size on mental health has provided mixed findings [2]. The immense interest into this topic has fuelled the production of a large research literature. Yet, despite this, results are conflicting and largely inconclusive [2, 3]. SNS are also constantly evolving; new networks join, while others lose in popularity [26], all while people’s networks tend to expand [21].

The aim of this study was to examine the association of SNS use, in itself, and in relation to “offline *PES*”, with *mental health* of young adults aged 18–34 in Sweden. More specifically, the questions are:

a) Does an association exist between *frequency of SNS use* or *number of SNS contacts* with *mental health* of young adults in Sweden?

b) Are young adults living in Sweden who have a high level of *PES* protected from the potentially detrimental effects of *frequent SNS use* or having a large *number of SNS contacts*?

## Methods

### Design, participants and setting

The 2016 Scania Public Health Survey comprised individuals between 18 and 96 years of age. However, as most previous studies concerned younger populations, we decided to restrict this study to “young adults” (i.e. individuals between 18 and 34 years of age). The main reason for this was that the prevalence of high SNS use has been shown to be considerably lower among older individuals [4], which would invite a problem of statistical power. Thus, our sample comprised 1341 individuals (477 males and 864 females) (Table 1). A postal questionnaire has been sent out regularly in about 5-year intervals, collecting data on health, employment, environment, and more, from randomly chosen individuals 18–80 years of age residing in the county of Scania, southern Sweden [27]. In 1999, a cohort of the 13,589 individuals who responded to the survey was created, with young individuals being added to the cohort in 2010 and 2016. In the most recent survey in 2016, items tapping Internet behaviour were added. The individuals used in this study were between 5 and 21 years old when Facebook was made available to anyone with an e-mail address (accessibility beyond solely universities), provided they were aged 13 or above [28].

**Table 1:** Distribution of sociodemographic characteristics, social networking site (SNS) use, social characteristics and poor mental health

Variables (missing values)	Category	Male		Female	
		ns	% of valid total	ns	% of valid total
Age group	18–26	263	55.1	495	57.3
	27–34	214	44.9	369	42.7
Poor mental health	No	300	62.9	435	50.3
	Yes	177	37.1	429	49.7
Frequency of SNS use (4)	Not at all	49	10.3	35	4.1
	Max. a couple times/ month	23	4.8	20	2.3
	A few times per week	33	6.9	34	3.9
	At least once every day	78	16.4	147	17.1
	Several times each day	222	46.7	452	52.4
	Almost every hour	70	14.7	174	20.2
Number of SNS contacts (39)	0–299	256	56.3	472	55.7
	300–599	142	31.2	273	32.2
	600–999	57	12.5	102	12.0
Main occupation (18)	Working	282	60.3	466	54.5
	Student	140	29.9	262	30.6
	Other	46	9.8	127	14.9
Education (17)	13 yrs. or more	262	55.6	558	65.4
	12 yrs. or less	209	44.4	295	34.6
Relationship status (22)	Single	190	40.6	286	33.6
	Married/ cohabiting with partner	211	45.1	444	52.2
	Other type of partnership	67	14.3	121	14.2
Born in Sweden (7)	Yes	415	87.4	768	89.4
	No	60	12.6	91	10.6
Perceived emotional support (5)	Yes, for sure	327	68.7	637	74.1
	Yes, probably	102	21.4	164	19.1
	Not quite sure	33	6.9	41	4.8
	No	14	2.9	18	2.1

Poor mental health is defined by the GHQ-12. Results represent the total population of participants aged 18–34 (*N* = 1341 (477 male and 864 female)) and are presented by gender. Data are gained from the Scania Public Health Cohort 2016

### Measures and variables

*Mental health* was assessed using the 12-item General Health Questionnaire (GHQ-12). The GHQ-12 aims to detect psychiatric morbidity [29] and is intended for screening non-specific psychiatric illnesses of a non-severe nature [30]. Though it is aimed at detecting minor mental distress and disorders in general, the test tends to be mostly indicative of depressive symptoms [31]. Each item has four responses, generally reading “better than usual”, “the same as usual”, “worse than usual” and “much worse than usual”. At least 8/12 questions had to be answered to be counted as a valid GHQ assessment. The Standard/GHQ scoring method (0–0–1–1) was utilised (range 0–12), as recommended by the creators of the instrument, with being a “GHQ-case” defined as a score of 2 or higher [32, 33]. The GHQ-12 scale has been found to be excellent for distinguishing depressive participants from healthy controls of a Swedish adult general population when using the GHQ scoring method, with sensitivity and specificity scores of 85.5 and 83.2, respectively [34].

*Frequency of SNS use* was measured using a single-item categorical scale to assess the extent of activity on SNS, such as Facebook, Twitter, Instagram and so on. Participants indicated their frequency of use during the last 12 months from the categories, “not at all”, “maximum a couple times per month”, “a few times per week”, “at least once per day”, “several times each day” and “almost every hour”. This variable was, based on its distribution, dichotomised to “almost every hour” and “less than almost every hour”.

*Number of SNS contacts* was measured using a single-item continuous scale, in which participants entered “Approximately how many friends or contacts they have in social media” in 3 available boxes, therefore answers range from 0 to 999 contacts. This item was also dichotomised based on its distribution to ≤599 or ≥ 600 contacts.

*Perceived emotional support* was assessed using a single item phrased “Do you feel that you have anyone or some people that can give you real personal support to manage life’s stresses and problems?”. Answers included “Yes, for sure”, “Yes, probably”, “Not quite sure” and “No”, which were dichotomised to High and Low, whereby Low included the latter three answers. The question encompasses social support received from any person, be it family, friends or significant others [24].

*Several background variables* were considered to potentially influence the true result. Age was kept as a continuous variable in analyses but was dichotomized by the median to groups aged 18–26 and 27–34 in Tables 1 and S1 (Supplementary file 1). Gender was defined as “male” or “female”. Relationship status included “Married to or cohabiting with partner”, “Single” and “Other type of partnership”, whereby the latter may be a long-distance partnership, for example. This variable was dichotomised to “Married to or cohabiting with partner” and “Single or Other type of partnership”. Current main occupation included categories “Working”, “Student” and “Other”, whereby “Student and Other” was combined to one category. Working persons included all that worked either full time or part-time (due to being partially on sick leave, unemployed or retired). Students included all persons studying, whether they were also employed or not. The “other” category included “Labour market action”, e.g. “trainee”, “completely unemployed”, “home-maker without economic reimbursement”, “retired full-time”, “long-term sick leave” and “parental leave”.

### Statistical methods and data analysis

The relationships between background factors and mental health are presented as numbers, frequencies and odds ratios. Further, logistic regression analyses were performed, using the IBM SPSS programme version 25. An alpha level of ≤5% was required for statistical significance. For the two multivariate models, several background variables were logically considered to be potential confounders, such as gender, age, educational level, current main occupation, relationship status, born in Sweden and PES [35]. If statistically significant, the variables were added in a stepwise manner to the regression models (Tables S2 and S3, see Supplementary files 2 and 3, respectively) [36]. Age as a continuous variable was not a significant predictor to the outcome, although when dichotomised and categorised in the female regression analysis, some significantly increased odds was found for the 18–26 age group (Table S1, Supplementary file 1). However, the primary reason for keeping age in the model was for comparability purposes. The gender variable was further analysed by splitting the file, whereby it became clear that the association between exposure and outcome was only evident in females. All subsequent analyses were therefore conducted separately for gender. The variables main occupation, relationship status and PES were all statistically significantly associated with mental health, and they were thus included in the logistic regression models.

Possible effect modification by PES on the association between the two main exposures and mental health was analysed using Rothmans method for creating interaction terms [37], for the females only. Synergy index was calculated using the following equation [37]:

$$ {\left(\mathrm{OR}-1\right)}_{\mathrm{EXP}++}/\left({\left(\mathrm{OR}-1\right)}_{\mathrm{EXP}+-}+{\left(\mathrm{OR}-1\right)}_{\mathrm{EXP}-+}\right). $$

## Results

### Descriptive statistics

Table 1 shows the sociodemographic data for the population investigated. More females (*n* = 864 (64.4%)) partook in the survey than males (*n* = 477 (35.6%)). Half the female population considered themselves to have poor mental health (49.7%) compared to males with a comparatively lower prevalence (37.1%). Around half the population use SNS several times every day (46.7 and 52.4% for men and women, respectively). The proportion of females that reported that they did not use SNS at all, was considerably lower (only 4.1%) compared to males (10.3%). The number of SNS contacts distribution is very much equal across genders, but most participants have under 300 friends. Having low PES is generally less common, though slightly more prevalent among men (31.2% for men vs. 26.0% for women).

### Research question a) SNS exposures and mental health

Cross-tabulations and regression analyses in Table S1 (see Supplementary file 1) showed more detailed discrepancies between genders, in that poor mental health prevalence in the “Almost every hour” category increased for women (62.6%) and remained relatively stable for men (38.6%) compared to the reference group. The same prevalence pattern was seen in the number of SNS contacts between genders. Women who reported using SNS almost every hour had an odds ratio (OR) of 1.94 (CI = 1.38–2.73) concerning GHQ case status compared to those that reported using SNS less than almost every hour. Similarly, women with ≥600 SNS contacts had an OR of 1.79 (CI = 1.17–2.74) regarding poor mental health compared to women who reported having fewer than 600 contacts. The mean number of SNS contacts for women was 288 (standard deviation (SD) = 223) and for men 284 (SD = 246). PES was highly associated with being a GHQ-case, whereof only a slight difference between genders was noticeable.

Tables S2 (see Supplementary file 2) and S3 (see Supplementary file 3) show the multivariate logistic regression models for the two relationships of interest, namely frequency of SNS use and number of contacts with poor mental health, respectively. For females, the independent effects of association between frequency of SNS use and poor mental health remained strong (OR 1.66 (CI = 1.16–2.38)) in the fully adjusted model (Model 3). A slight decreasing trend occurred after stepwise adjustment (Models 1, 2 and 3). However, the only other significantly associated exposure variable was PES (OR 1.96 (CI = 1.42–2.71)), whereas age, main occupation and relationship status were not. The same pattern was seen in Table S3 (see Supplementary file 3)**,** which demonstrates that the fully adjusted Model 3 association between number of SNS contacts and poor mental health remained statistically significant (OR 1.89 (CI = 1.21–2.97)). Neither of the associations of interest (Tables S2 and S3, Supplementary files 2 and 3) were much affected by the potentially confounding variables. For males there was no association demonstrated in Model 3 concerning any of the two main exposure variables, after full adjustment of the chosen covariates (Table S2: OR 0.94 (CI = 0.54–1.66), and Table S3: OR 0.96 (CI = 0.52–1.76)).

### Research question b) PES and mental health

This section builds on the first research question by aiming to detect how PES moderates the effect of frequency of SNS use and the number of SNS contacts regarding mental health. Provided the non-existing bivariate association in males, the subsequent analysis was not included for them.

Table 2 shows that among those who reported being active on SNS almost every hour, the effect on mental health was highest for those who simultaneously reported low PES, OR 3.12 (CI = 1.69–5.76). However, the evidence for significant effect modification between the mentioned exposure variables was weak, SI = 1.25.

**Table 2:** Synergistic interaction effects between SNS use frequency and PES on poor mental health among females

Category	Total N (% of valid total)	Cases N (% within category)	OR (95% CI)
Less than almost every hour & high PES	506 (60.4)	216 (42.7)	1 (ref)
Almost every hour & high PES	115 (13.7)	67 (58.3)	1.70 (1.12–2.59)*
Less than almost every hour & low PES	162 (19.3)	98 (60.5)	2.00 (1.39–2.87)*
Almost every hour & low PES	55 (6.6)	39 (70.9)	3.12 (1.69–5.76)*
Total	838 (100)^a^	420 (50.1)	SI = 1.25

Results are presented as total numbers, numbers and percentages of cases of poor mental health within each category, odds ratios (OR) with 95% confidence intervals (95% CI), and the synergy index (SI). Interactions were controlled for age, main occupation and relationship status. Data are gained from the Scania Public Health Cohort 2016. Total number of female Swedish young adults = 838^a^. a: Missing values = 26 (3.0%). * Significant result = *p* < 0.05

Similarly, Table 3 shows that among those who reported having ≥600 contacts on SNS, the effect on mental health was highest among those who at the same time reported having low PES, OR 6.07 (CI = 1.73–21.33). In this case, there was an indication that the level of PES modified the effect of having many SNS contacts on mental health, so that low PES reinforced the negative effect of having a large number of contacts, SI = 2.88.

**Table 3:** Synergistic interaction effects between number of SNS contacts and PES on poor mental health among females

Category	Total N (% of valid total)	Cases N (% within category)	OR (95% CI)
0–599 contacts & high emotional support	530 (64.2)	233 (44.0)	1 (ref)
600–999 contacts & high emotional support	80 (9.7)	48 (60.0)	1.75 (1.08–2.85)*
0–599 contacts & low emotional support	198 (24.0)	122 (61.6)	2.01 (1.43–2.81)*
600–999 contacts & low emotional support	18 (2.2)	15 (83.3)	6.07 (1.73–21.33)*
Total	826 (100)^b^	418 (50.6)	SI = 2.88

Results are presented as total numbers, numbers and percentages of cases of poor mental health within each category, odds ratios (OR) with 95% confidence intervals (95% CI), and the synergy index (SI). Interactions were controlled for age, main occupation and relationship status. Data are gained from the Scania Public Health Cohort 2016. Total number of female Swedish young adults = 826^b^. b: Missing values = 38 (4.4%). * Significant result = *p* < 0.05

## Discussion

The findings of this study indicate an association between high frequency of SNS use and a large network size on the one side and poor mental health on the other side, among Swedish young female adults. Furthermore, it shows that this association is modified by the level of PES. Women who used SNS on an almost hourly basis had increased odds of experiencing poor mental health than when using the platforms less frequently, as well as when having more than 600 contacts on SNS compared to fewer. Women with high PES seemed largely protected from the detrimental effects of having more than 600 SNS contacts.

Despite the widespread knowledge that women, especially young women [38], are more frequently burdened by depression than men [39], it seems somewhat surprising that very few studies have specifically looked into the difference of impact between genders in SNS use and mental health [2, 3].

### Early age gender difference

In a recent, longitudinal UK study, girls and boys aged 10–15 showed decreasing happiness and increasing active SNS usage [40]. Additionally, socio-emotional difficulties (emotional, peer-relationship and conduct problems) rose for girls but declined for boys. The increased use of SNS at age 10 was associated with decreased happiness and increased socio-emotional difficulties in later years thereafter (up to age 15), in girls only. This study showed how at a very young age, use of SNS can affect girls’ well-being in later adolescent years [40]. It is thought that since the adolescent years shape one’s future physical and mental health to a large extent [41], many adolescents experiencing mental health issues during this crucial developmental period will go on to experience such issues in later years, especially in their 20’s [42]. Hence, a decline in well-being due to SNS use occurring from an early age onwards, could escalate and result in mental health issues in later young adult years. However, it may be, that this trend reverses beyond the young adult years, as women aged 27–34 in this study were slightly less likely to indicate poor mental health (Table S1).

Another study of an adolescent population with a mean age of 15 years found that passive Facebook use increased depressed mood in girls only, whereas active, public (posting content on their profiles) Facebook use was associated with depressed mood in boys. However, active public and private use in girls yielded positive outcomes when they perceived online social support [43]. Together, these studies suggest that SNS use affects children already at a young age, that these effects vary by gender, and by the way in which the sites are used.

### Problematic Facebook use & a gender perspective

A meta-analysis on Problematic Facebook Use (PFU), or Facebook addiction, suggested that females were more prone to demonstrate PFU behaviour than males [44]. One definition of PFU is such Facebook usage that disrupts everyday life at school, work or with relationships, by causing distress in cognitive functioning and/or well-being [44]. In a meta-analysis of 23 studies with adolescent and young adult populations, PFU was positively associated with depression, anxiety and psychological distress in general [45]. Marino et al. (2018) found that time spent on Facebook correlated with PFU [44]. Females in the present study who reported using SNS almost hourly could in fact be described as exercising PFU behaviour. Of course, frequency of SNS use does not completely equate to time spent on Facebook, but the two variables go hand in hand. PFU individuals tended to have larger friendship networks compared to non-PFU individuals, and females with PFU also tended to send more friend requests and private messages [46]. It has been repeatedly shown that women use SNS for communicating, maintaining friendships and accessing social information, whereas men tend to use the platforms more for gaining information and playing games [47–49]. A deeper understanding as to why women use SNS more and are potentially also detrimentally affected by them could be provided by a gender perspective [50]. Essentially, gender roles are transposing onto the use of SNS, so that women are more attracted to use SNS for social connectivity than men [43, 47]. For example, the behaviour to compare oneself on SNS is more common among women than men [51]. Indeed, increased Facebook use has been linked to increased social comparison, fuelling envy, and resulting in depression, which is partially grounded in causal evidence [22]. A difference between genders has also been detected, in that adolescent females who tended to compare themselves on SNS had worse depression outcomes [52]. Interestingly, when the number of SNS contacts was analysed in this study as a continuous variable, there was a statistically significant association between each additional contact and being a case for poor mental health in the female population, with an incremental chance of 0.1%, OR 1.001 (CI = 1.00–1.001). Though a small value, it is nonetheless meaningful, considering the number of contacts are as many as several hundred for many SNS users. Also, females have been shown to emotionally respond worse to negative images or messages, which may provide further explanation for the poorer health outcomes [50].

Our finding regarding the gender difference in the association between number of SNS contacts and mental health appears to be the first such finding reported in the scientific literature.

### No gender differences

Some studies do not show differences in gender, such as Kross et al. (2013) [10], where declines in subjective well-being, associated with frequent Facebook use, were not moderated by gender. Gender was also accounted for by Verduyn et al. (2015) [14], however it did not moderate the relationships [14]. Although SNS use was associated with body image and eating disorders, this meta-analytic association did not differ between men and women in studies that examined gender [53].

It may be that a true association between SNS use and mental health has been clouded by combining gender in analyses, contributing to the mixed findings in the literature. Although most studies controlled for gender, a deeper analysis as in the above studies (sections Early age gender difference and No gender differences) has been the exception.

### PES & SNS use

Women are, in the absence of emotional support, significantly more prone to depression than men [54]. This was corroborated by the findings in this study. Similarly, in another Swedish study of late adolescents, women benefitted more than men in their psychological well-being from high-quality, trusting friendships [55]. The findings in our study showed that high PES protected very frequent SNS users from a negative impact on their mental health. However, the current findings may be an artefact in that persons with low PES inherently use SNS more than individuals with high PES, as a meta-analytic study showed low PES led to loneliness, which subsequently led to increased Facebook use [16]. A further study, which aimed to investigate PFU, performed a 3-way interaction analysis between PFU, neuroticism and well-being, which differed according to gender. Women high in PFU and neuroticism were at 17 times higher odds of having low mood compared to men low in neuroticism and PFU [56]. It has been shown that neuroticism and PES are related, in that females high in neuroticism also perceive lower emotional support [57], suggesting a similar finding to ours regarding the interaction between frequency of SNS use and low PES.

Most PES inquiries are concerned with online-derived support rather than offline ditto [2]. For example, one such inquiry found that larger Facebook networks were associated with increased perceived online social support as well as life satisfaction [21]. Further analysis in the above-mentioned study found a negative curvilinear relationship between the number of SNS contacts and perceived social support [58]. This meant that *from* a certain number and *up to* a certain number of Facebook friends, these provided perceived social support, but beyond these points, no or too little support was derived; the authors suggest this may be due to the time and effort devoted to friends becoming too much or too little, thereby contributing detrimentally [58]. Further comparable studies are sparse, so that the finding of effect modification by PES on the association between SNS use and mental health appears to be a novel finding.

### Methodological considerations & limitations

The main limitation of this study is the cross-sectional design, which does not exclude causal effects in both directions between the main variables, and it is not unlikely that the causal mechanism may be bidirectional. Since non-response was not negligible, selection bias could be an issue and theoretically leading to either overestimation or underestimation of the found associations. However, it seems unlikely to be the major explanation of the findings. A strength of this study is the comparatively large sample size, which reduces the risk of random error. The main exposure variables were developed by one of the authors due to lack of well-validated alternatives, and are rather straight-forward questions on frequency of SNS use and number of contacts which are unlikely to be misunderstood by the respondents or to be an issue of other types of response bias. Since self-reported SNS use may be under-reported when compared to actual use, there is a risk of differential misclassification (i.e. if some high users with poor mental health were erroneously classified as non-high-users), and thus, our findings may represent an under-estimation of the true effect [59]. The outcome variable was measured by a very well-validated instrument, which also reduces the likelihood of misclassification. The associations were controlled for the most obvious potential confounders: age, occupation, relationship status, as well as PES, which does not exclude bias from confounding, but ought to render it a less likely explanation of the found associations. Finally, the SNS measures used in this study were not specific to certain SNS. As these sites vary in their purpose of usage, interface and content, it would be beneficial to include separate SNS survey questions. Facebook is the most commonly studied SNS [2], hence also the literature presented here is biased towards Facebook. Having said that, most Swedes use Facebook [4], therefore it is probable that these results are most applicable to Facebook.

## Conclusions

Important findings emerged, in that statistically significant associations between frequency of SNS use and number of SNS contacts on one side, and mental health on the other side were found – albeit only among female participants. Furthermore, that this association was modified by the level of PES.

From this study’s findings, it is suggested that future research conducts deeper gender analyses, whether through stratifying by gender, conducting separate analyses or limiting the study population. These findings also raise further questions, hence why it is necessary to deeply investigate how SNS are used by women compared to men, allowing for comparability and providing information on protective behaviours. Equally important is to investigate the possibility of a causal association by employing more longitudinal studies.

In light of this study, SNS use in women with mental health issues needs to be targeted. For instance, psychological strategies to enhance young women’s PES could be utilised in therapy, at institutions (universities, schools) and within communities. Spreading understanding of potential detrimental effects of frequent SNS use or having relatively large SNS networks, and how to establish healthy SNS usage patterns, as well as form a SNS network that works to one’s benefit, may be target areas with which to improve mental health outcomes.

## Supplementary Information

**Supplementary file 1**: **Table S1.** Sociodemographic characteristics, SNS use, and social characteristics, in relation to poor mental health.

**Supplementary file 2**: **Table S2.** Logistic regression showing the associations between frequency of SNS use and poor mental health.

**Supplementary file 3**: **Table S3.** Logistic regression showing the associations between number of SNS contacts and poor mental health.

## Data Availability

The datasets generated and/or analysed during the current study are available by request to the corresponding author.

## Abbreviations

SNS: Social networking site
PES: Perceived emotional support
GHQ: General health questionnaire
