# Supplementary material for: Investigating the effect of social networking site use on mental health in an 18–34 year-old general population; a cross-sectional study using the 2016 Scania Public Health Survey
Source: BMC Public Health. 2020 Nov 23;20:1753. doi: 10.1186/s12889-020-09732-z (PMC7682097; doi:10.1186/s12889-020-09732-z)
Supplement: Supplementary file 1 — Table S1. Sociodemographic characteristics, SNS use, and social characteristics, in relation to poor mental health. [file 12889_2020_9732_MOESM1_ESM.docx]

**Supplementary file 1**

**Table S1.
Sociodemographic characteristics, SNS use, and social characteristics, in relation to poor mental health.**

| Variables | Categories | Male | | Female | |
| --- | --- | --- | --- | --- | --- |
|  |  | ns total /  ns of cases (%) | OR (95% CI) | ns total /  ns of cases (%) | OR (95% CI) |
| Age | 27–34 | 214 / 76 (35.5) | 1 (ref) | 369 / 168 (45.5) | 1 (ref) |
|  | 18–26 | 263 / 101 (38.4) | 1.13 (0.78–1.65) | 495 / 261 (52.7) | 1.33 (1.02–1.75)* |
| Frequency of SNS use | Less than almost hourly | 405 / 149 (36.8) | 1 (ref) | 688 / 319 (46.4) | 1 (ref) |
|  | Almost every hour | 70 / 27 (38.6) | 1.08 (0.64–1.82) | 174 / 109 (62.6) | 1.94 (1.38–2.73)* |
| Nu. of SNS contacts | 0–599 | 398 / 149 (37.4) | 1 (ref) | 745 / 361 (48.5) | 1 (ref) |
|  | 600–999 | 57 / 23 (40.4) | 1.13 (0.64–1.99) | 102 / 64 (62.7) | 1.79 (1.17–2.74)* |
| Main Occupation | Working | 282 / 88 (31.2) | 1 (ref) | 466 / 215 (46.1) | 1 (ref) |
|  | Student and Other | 186 / 83 (44.6) | 1.78 (1.21–2.61)* | 389 / 212 (54.5) | 1.40 (1.07–1.83)* |
| Relationship Status | Married to/ cohabiting with partner | 211 / 67 (31.8) | 1 (ref) | 444 / 208 (46.8) | 1 (ref) |
|  | Single/ Other type of partnership | 257 / 104 (40.5) | 1.46 (0.997–2.14) | 407 / 216 (53.1) | 1.28 (0.98–1.68) |
| Born in Sweden | Yes | 415 / 151 (36.4) | 1 (ref) | 768 / 386 (50.3) | 1 (ref) |
|  | No | 60 / 24 (40) | 1.17 (0.67–2.03) | 91 / 41 (45.1) | 0.81 (0.52–1.26) |
| PES | High | 327 / 106 (32.4) | 1 (ref) | 637 / 287 (45.1) | 1 (ref) |
|  | Low | 149 / 71 (47.7) | 1.90 (1.28–2.82)* | 223 / 140 (62.8) | 2.06 (1.50–2.81)* |

*Results are presented by gender, with total numbers, numbers and percentages of cases of poor mental health within each category, and finally, with unadjusted odds ratios (OR) with 95% confidence intervals (95% CI). Scania Public Health Cohort 2016; N = 1341 (477 male and 864 female). * Significant result = p<0.05.*
