# Supplementary material for: Investigating the effect of social networking site use on mental health in an 18–34 year-old general population; a cross-sectional study using the 2016 Scania Public Health Survey
Source: BMC Public Health. 2020 Nov 23;20:1753. doi: 10.1186/s12889-020-09732-z (PMC7682097; doi:10.1186/s12889-020-09732-z)
Supplement: Supplementary file 2 — Table S2. Logistic regression showing the associations between frequency of SNS use and poor mental health. [file 12889_2020_9732_MOESM2_ESM.docx]

**Supplementary file 2**

**Table S2.**

**Logistic regression showing the associations between frequency of SNS use and poor mental health.**

| Variable | Category | Male | | | Female | | |
| --- | --- | --- | --- | --- | --- | --- | --- |
|  |  | Model 1^a^ | Model 2^b^ | Model 3^c^ | Model 1^a^ | Model 2^b^ | Model 3^c^ |
| Frequency of SNS use | Almost every hour (vs less than almost every hour) | 0.99 (0.58–1.72) | 0.91 (0.52–1.59) | 0.94 (0.54–1.66) | 1.87 (1.32–2.64)* | 1.74 (1.12–2.48)* | 1.66 (1.16–2.38)* |
| Main Occupation | Student/Other (vs Working) |  | 1.92 (1.24–2.99)* | 1.86 (1.19–2.91)* |  | 1.30 (0.98–1.74) | 1.29 (0.96–1.72) |
| Relationship Status | Single/other partner (vs married/cohabiting) |  | 1.59 (1.04–2.45)* | 1.49 (0.96–2.30) |  | 1.15 (0.85–1.55) | 1.09 (0.81–1.48) |
| PES | Low (vs high) |  |  | 1.69 (1.12–2.56)* |  |  | 1.96 (1.42–2.71)* |

*Results are presented by gender, as odds ratios with 95% confidence intervals. Scania Public Health Cohort. N = 1297 (459 male and 838 female). ^a^ Model 1: Unadjusted; ^b^ Model 2: Model 1 + adjusted for age, main occupation and relationship status; ^c^ Model 3: Model 2 + adjusted for PES. * Significant result = p<0.05.*
