# Supplementary material for: Investigating the effect of social networking site use on mental health in an 18–34 year-old general population; a cross-sectional study using the 2016 Scania Public Health Survey
Source: BMC Public Health. 2020 Nov 23;20:1753. doi: 10.1186/s12889-020-09732-z (PMC7682097; doi:10.1186/s12889-020-09732-z)
Supplement: Supplementary file 3 — Table S3. Logistic regression showing the associations between number of SNS contacts and poor mental health. [file 12889_2020_9732_MOESM3_ESM.docx]

**Supplementary file 3**

**Table S3.**

**Logistic regression showing the association between number of SNS contacts and poor mental health.**

| Variable | Category | Male | | | Female | | |
| --- | --- | --- | --- | --- | --- | --- | --- |
|  |  | Model 1^a^ | Model 2^b^ | Model 3^c^ | Model 1^a^ | Model 2^b^ | Model 3^c^ |
| Number of SNS contacts | 600–999 contacts (vs 0–599) | 0.99 (0.55–1.78) | 0.90 (0.50–1.65) | 0.96 (0.52–1.76) | 1.89 (1.22–2.93)* | 1.74 (1.12–2.72)* | 1.89 (1.21–2.97)* |
| Main Occupation | Student/Other (vs Working) |  | 1.94 (1.24–3.03)* | 1.85 (1.18–2.90)* |  | 1.30 (0.97–1.74) | 1.28 (0.96–1.72) |
| Relationship Status | Single/other partner (vs married/cohabiting) |  | 1.56 (1.01–2.41)* | 1.47 (0.95–2.29) |  | 1.12 (0.83–1.51) | 1.05 (0.77–1.43) |
| PES | Low (vs high) |  |  | 1.73 (1.13–2.64)* |  |  | 2.08 (1.51–2.88)* |

*Results are presented by gender, as odds ratios with 95% confidence intervals. Scania Public Health Cohort. N = 1268 (442 male and 826 female). ^a^ Model 1: Unadjusted; ^b^ Model 2: Model 1 + adjusted for age, main occupation and relationship status; ^c^ Model 3: Model 2 + adjusted for PES. * Significant result = p<0.05.*
